# The Role of Stress in Alcohol Use, Alcoholism Treatment, and Relapse

**Published:** 1999

**Authors:** Kathleen T. Brady, Susan C. Sonne

**Affiliations:** Kathleen T. Brady, M.D., Ph.D., is a professor of psychiatry, and Susan C. Sonne, Pharm.D., is an assistant professor of psychiatry and pharmacy at the Medical University of South Carolina, Charleston, South Carolina

**Keywords:** psychological stress, causes of AODU (AOD use, abuse, and dependence), addiction care, AODD (AOD use disorder) relapse, neurobiological theory, animal model, AOD use initiation, AOD use susceptibility, AOD abstinence, AODU development, stress management skills, drug therapy, psychosocial treatment method, literature review

## Abstract

Addiction to alcohol or other drugs (AODs) is a complex problem determined by multiple factors, including psychological and physiological components. Stress is considered a major contributor to the initiation and continuation of AOD use as well as to relapse. Many studies that have demonstrated an association between AOD use and stress have been unable to establish a causal relationship between the two. However, stress and the body’s response to it most likely play a role in the vulnerability to initial AOD use, initiation of AOD abuse treatment, and relapse in recovering AOD users. This relationship probably is mediated, at least in part, by common neurochemical systems, such as the serotonin, dopamine, and opiate peptide systems, as well as the hypothalamic-pituitary-adrenal (HPA) axis. Further exploration of these connections should lead to important pharmacological developments in the prevention and treatment of AOD abuse. Studies indicate that treatment techniques which foster coping skills, problem-solving skills, and social support play a pivotal role in successful treatment. In the future, individualized treatment approaches that emphasize stress management strategies in those patients in whom a clear connection between stress and relapse exists will become particularly important.

Clinicians and researchers consider the addiction to alcohol or other drugs (AODs) a complex problem determined by multiple factors, including psychological and physiological components. Many theories involving numerous variables (e.g., personality and access to AODs) have sought to explain the initiation and maintenance of AOD abuse and dependence. Most of those theoretical models consider stress a major contributor to the initiation and continuation of AOD use as well as to relapse. Accordingly, the relationship between stress and alcohol use has received much attention, as evidenced by the articles in this issue of *Alcohol Research & Health.*

The notion that exposure to stress-inducing factors in everyday life (i.e., life stressors) can cause susceptible people to initiate or relapse to alcohol use has intuitive appeal. (For more information on stress and the body’s response to stress, see sidebar, p. 264.) Whereas the relationship between stress and AOD use can be studied fairly easily in laboratory animals, a definitive exploration of this connection in humans has been more difficult. Animal studies generally have supported the positive relationship between stress and alcohol use and abuse. Researchers also have begun to focus on an organism’s response to stress and the consequences of AOD use and how it affects biological processes in the brain. These studies have identified several neurobiological connections between the changes produced by stress and the changes produced by both short-term (i.e., acute) and long-term (i.e., chronic) AOD use (Piazza and Le Moal 1998).

**Stress and the Body’s Response**

The term “stress” generally refers to the reactions of the body to certain events or stimuli that the organism perceives as potentially harmful or distressful. Such stress-inducing events or stimuli, which are referred to as stressors, can be either physical (e.g., unusual environmental conditions or a physical attack) or psychological (e.g., occupational or familial difficulties) in nature. Individual people respond differently to different stressors. An event that is perceived as extremely stressful by one person may be perceived as harmless by another.

Whenever an organism perceives a situation as stressful, it initiates a stress response—that is, a complex spectrum of behavioral reactions, such as escape or avoidance behaviors; biological reactions, such as increases in heart rate, blood pressure, or sweating; and (in humans) emotional reactions, such as feelings of anxiety.

The stress response is coordinated through two mechanisms: (1) changes in the activities of various brain regions and brain chemicals (i.e., neurotransmitters) and (2) changes in the activity of a hormonal system called the hypothalamic-pituitary-adrenal (HPA) axis (see figure 1, p., 266). Neurotransmitters involved in controlling the stress response include serotonin, dopamine, and opioid peptides. These neurotransmitter systems act through a variety of mechanisms. Thus, opioid peptides directly lead to pain relief, and dopamine release results in increases in blood pressure and heart rate. Furthermore, various neurotransmitters can affect the body more indirectly by inhibiting or enhancing the activity of the HPA axis.

The HPA axis consists of three hormones: (1) corticotropin-releasing hormone (CRH), which is produced in a brain region called the hypothalamus; (2) adrenocorticotropic hormone (ACTH), which is released from the pituitary gland located below the hypothalamus; and (3) glucocorticoid hormones, which are secreted from the adrenal glands located on top of the kidneys. The three types of hormones form a tightly regulated hormone cascade. Activation of various nerve cells (i.e., neurons) in the brain in response to stress results in the production and release of CRH from certain cells in the hypothalamus. Through specific blood vessels in the brain, CRH is transported from the hypothalamus to the pituitary gland, where it induces the production and secretion of ACTH into the body’s general circulation. Through the blood, ACTH reaches the adrenal glands and initiates the production and release of glucocorticoid hormones. (The major glucocorticoid in humans is cortisol, whereas the major glucocorticoid in rodents is corticosterone.) These glucocorticoids induce and regulate the body’s diverse physiological responses to stress, such as changes in cardiovascular function and sweat gland activity.

The activity of the HPA axis is regulated by a negative feedback mechanism in which glucocorticoids released into circulation act back on the hypothalamus and/or pituitary gland to suppress further release of CRH and/or ACTH.

The body’s neurochemical and hormonal responses to stress do not act independently of each other, but are tightly interconnected. Thus, CRH release in the hypothalamus is regulated by neurons releasing serotonin or endogenous opioids. Furthermore, CRH release not only results in ACTH release, but also in the release of certain endogenous opioids from specific neurons in the brain, which may contribute to various behavioral and emotional consequences of stress.

—Kathleen T. Brady and Susan C. Sonne

In the clinical arena, however, the relationship between stress and alcohol use has been more difficult to characterize. For example, human laboratory studies have not uniformly supported a prominent theory called the tension-reduction hypothesis of alcohol use, which posits that people use alcohol to reduce stress. Furthermore, studies of the relationship between stress and alcohol use are difficult to conduct in alcoholic patients and, as a result, have numerous inherent limitations. Study participants may recall only selective events that have contributed to alcohol use, may be inconsistent about which events to include as stressors, and may have difficulties distinguishing between events that precipitate alcohol use and those that result from alcohol use and relapse.

Because of these difficulties, many studies that have demonstrated an association between AOD use and stress have been unable to establish a causal relationship between the two. For example, heavy alcohol users frequently experience stress related to occupational, social, legal, and financial problems. When interpreting such observations, some investigators have chosen to classify stressful events as illness dependent or illness independent, depending on whether they are caused by the AOD use. This classification has not been consistently adopted, however, and many studies fail to determine the degree to which such stressors occur independent of alcohol use, cause alcohol use, or are a consequence of alcohol use.

The type of stressor studied also influences analyses of the relationship between stress and alcohol use. For example, many studies investigating the role of stress in relapse after treatment have limited their focus to stressors that occurred after treatment completion. Some stressful life events that affect the lives of alcoholics after treatment, however, may have occurred before treatment (e.g., a divorce or job loss). Moreover, stressors can range from dramatic and severe events (e.g., a divorce or death of a loved one) to chronic irritants of daily life (e.g., job hassles or financial worries). Both the temporal relationship between stress and alcohol use and the type of stressor studied, however, can profoundly affect study results.

This article explores the relationship between stress and alcohol use, alcoholism treatment, and relapse. The discussion includes the results of animal studies that investigate the neurochemical and theoretical relationships between stress and the initiation or resumption (i.e., reinstatement) of alcohol use after abstinence. The article also considers the results of clinical and naturalistic studies^1^ that explore the connection between stress and AOD use in humans. Finally, the article reviews the role of stress management and stress reduction techniques in alcoholism treatment.

## Neurobiological Connections Between Stress and Addiction

**Figure 1:**
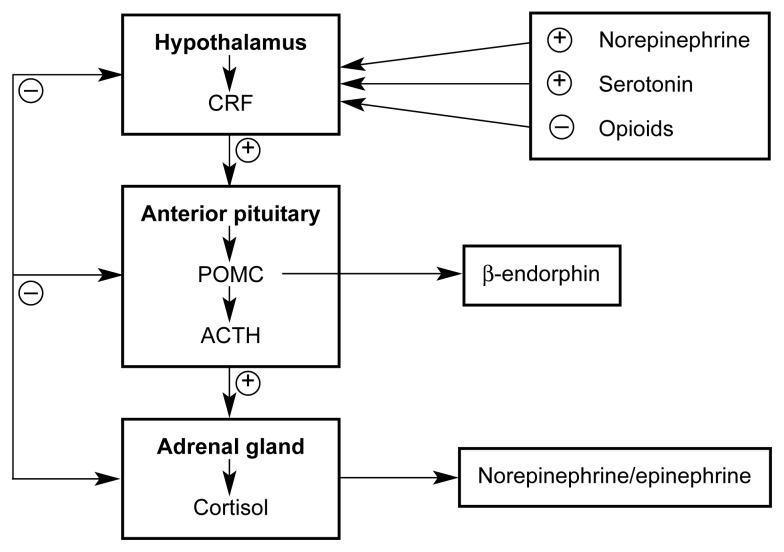
The hormone activity of the hypothalamic-pituitary-adrenal (HPA) axis. For clarity, the brain and body regions that produce the hormones appear in bold. NOTE: ACTH = adrenocorticotropic hormone; CRF = corticotropin-releasing factor; POMC = proopiome-lanocortin.

Animal studies have suggested that exposure to stress facilitates both the initiation and the reinstatement of AOD use after a period of abstinence (Kreek and Koob 1998). To better understand the biological basis of the effects of stress on AOD self-administration in animals, researchers have focused primarily on two neurobiological systems. The first system involves the organism’s hormonal and subsequent biological responses to stress and the influence of those responses on the reinforcing effects of AODs. Those studies, which aim mainly to identify specific, stress-induced hormonal changes that mediate the effects of stress on AOD self-administration, primarily have examined the activity of a hormone system called the hypothalamic-pituitary-adrenal (HPA) axis. (For more information on the HPA axis and its function, see sidebar, p. 264, and figure 1, p. 266.) This hormone system has three components:

- Corticotropin-releasing hormone (CRH), which is produced in a brain region called the hypothalamus
- Adrenocorticotropic hormone (ACTH), which is produced in the pituitary gland located in the brain below the hypothalamus
- Glucocorticoid hormones, such as cortisol in humans and corticosterone in rodents, which are produced in the adrenal glands that are located on top of the kidneys.

Glucocortocoid secretion by the adrenal gland is considered one of the central biological responses to stressful events (Piazza and Le Moal 1998). Studies have shown that both acute stress and alcohol or cocaine administration can activate the HPA axis, probably by acting on CRH. (Also see the article in this issue by Spencer and Hutchison, pp. 272–283.) Consistent with this hypothesis, agents that interfere with CRH function also decrease sensitivity to environmental stress in animal models and prevent some of the reinforcing effects of cocaine (Kreek and Koob 1998).

**Figure 2:**
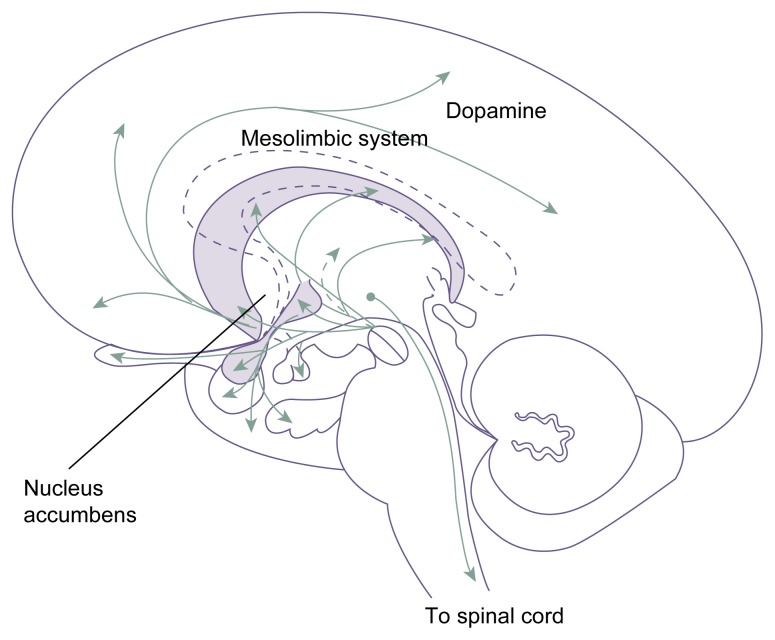
Dopaminergic pathways in the brain. Dopamine modulates such varied functions as emotion, aggression, cognition, the coordination of movement, and aspects of the development of addiction. SOURCE: Adapted from Heimer, L. *The Human Brain and Spinal Cord: Functional Neuroanatomy and*

The second neurobiological system investigated in animal studies of stress and AOD use involves the stress-induced changes in the activity of certain brain regions and brain molecules (i.e., neurotransmitters) assumed to play a role in mediating the reinforcing effects of AODs. This approach is based on the hypothesis that stress facilitates AOD self-administration in laboratory animals and humans by enhancing the activity of those neurobiological systems. This research has focused mostly on nerve cells (i.e., neurons) that are located in the midbrain (i.e., mesencephalon) and which use the neurotransmitter dopamine. Some of these neurons extend to the nucleus accumbens, which is considered one of the primary brain areas involved in mediating the reinforcing effects of various AODs (see figure 2, p. 267).

One likely explanation for the connection between stress and AOD use is that stress modifies the motivational and/or reinforcing effects^2^ of AODs at the neurobiological level. For example, stress increases the activity of the dopaminergic brain systems that are involved in motivation and reward and which also mediate AOD-induced rewarding effects. Accordingly, stress-induced changes in those systems could enhance the organism’s responsiveness to the effects of AODs. Furthermore, when an organism is in a stressful situation, numerous biological systems are activated to help the organism cope with the stress. For example, the adrenal glands release epinephrine to prepare the organism for a “fight or flight” response, and various brain regions secrete pain-relieving chemicals. Similarly, stress possibly results in increased activity in the dopaminergic system in an attempt to counteract the negative emotional state associated with stress (Piazza and Le Moal 1998).

Animal studies have suggested that another neurotransmitter, serotonin, also may play a role in the relationship between stress and AOD use. For example, alcohol administration increases brain serotonin metabolism in animals (LeMarquand et al. 1994). Furthermore, increases in serotonin levels and metabolism have been shown to decrease alcohol consumption in experimental animals (LeMarquand et al. 1994). Studies in nonhuman primates found that animals with low brain serotonin activity are high consumers of alcohol (Higley et al. 1998). When these high alcohol-consuming animals were treated with an agent that prevents serotonin breakdown and thus prolongs serotonin’s activity in the brain (i.e., a selective serotonin reuptake inhibitor [SSRI]), their alcohol consumption declined substantially. Clinical trials investigating the use of SSRIs in humans, however, have generated mixed results regarding the ability of those agents to decrease alcohol consumption.

In addition, animal studies have indicated that the brain’s serotonin systems also affect the brain regions that mediate another stress-related reaction, the fear response (Maier and Watkins 1998). Consistent with this observation, many SSRIs have demonstrated powerful activity in the treatment of anxiety disorders in humans in addition to their antidepressant activity. This association of the serotonin system with both consummatory behaviors and anxiety states further supports the notion that a neurobiological connection exists between stress and AOD use and abuse.

## Animal Models of the Relationship Between Stress and Relapse

Numerous studies using various animal models have examined the relationship between stress and the initiation or the reinstatement of alcohol use after a period of abstinence. These studies are summarized in the following sections.

### Stress and the Initiation of Alcohol Use

Animal studies have demonstrated that exposure to both acute and repeated stress can increase an animal’s potential for initiating AOD self-administration as well as modify the amount and frequency of established AOD self-administration. However, this relationship appears to depend on the timing of the exposure to a stressor and of the AOD exposure. For acute stress to induce AOD administration, the stressful event and the AOD exposure must occur within a short interval.

For example, in experiments in which animals were exposed to acute stress by restraining them for a short period of time, AOD self-administration was facilitated only if the stressful situation preceded the AOD exposure by no more than 30 minutes (Shaham 1993). When the animals were exposed to stress repeatedly or for prolonged periods, however, the interval between the end of the stressful situation and the AOD exposure did not appear to influence AOD self-administration. Thus, in those instances the animals showed increased AOD self-administration regardless of whether the stressful experiences continued up to the AOD-use assessment or had ended weeks earlier (Shaham 1993). These observations indicate that repeated stress can induce long-lasting modifications in neural pathways, resulting in a drug-prone state that is independent of the actual presence of the stressor (Piazza and Le Moal 1998).

Although stressors that have a physical component, such as a mild electric shock to the feet or a pinch in the tail, can lead to increased AOD self-administration, such physical manipulations do not appear to be required for mediating stress effects. In fact, psychological stress alone can also increase drug self-administration. For example, rats that witnessed another animal receiving an electric shock exhibited increased self-administration of cocaine (Ramsey and Van Ree 1993). Similarly, enhanced AOD self-administration occurred in studies in which animals were exposed to stress in the form of social aggression by being placed in an unfamiliar group of animals while being protected from actual physical attacks by a screen grid (Piazza and Le Moal 1998).

### Stress and the Reinstatement of Alcohol Use

Stressful experiences also can contribute to the reinstatement of AOD use after a period of abstinence in animals with a history of AOD self-administration. For example, studies in rats found that a single stressful experience, such as a one-time electrical shock to the feet, induced resumption of drug use in animals that had been previously taught to self-administer cocaine or heroin (Shaham and Stewart 1996; Erb et al. 1996). This stress-induced reinstatement of AOD use is a well-documented phenomenon. In fact, exposure to stress is the most powerful and reliable experimental manipulation used to induce reinstatement of AOD use (Haleem 1996).

Animal models of the relationship between stress and alcohol relapse have employed not only animals with a history of alcohol self-administration that had undergone a prolonged period of abstinence but also animals with a history of alcohol dependence that were deprived of alcohol. Studies found that both alcohol-dependent and non-alcohol-dependent animals will increase their response for alcohol (compared to base-like levels) following a period of imposed deprivation (Heyser et al. 1996).

Researchers have used such an alcohol deprivation model in dependent rats to investigate the effects of two medications used to treat alcoholism in humans, naltrexone and acamprosate. Both of these agents interfere with the actions of the neurotransmitters involved in mediating stress and the reinforcing effects of alcohol. Thus, naltrexone blocks the actions of neurotransmitters called endogenous opioids (i.e., an opioid antagonist), and acamprosate likely interferes with the function of the neurotransmitter glutamate. In alcohol deprivation studies in rats, both agents prevented the increase in alcohol self-administration normally observed in animals that experience stress as a result of a period of forced abstinence (Kreek and Koob 1998). Similarly, opioid antagonists (e.g., naltrexone) prevented the increase in alcohol consumption observed in animals exposed to other types of stress (Volpicelli et al. 1992).

Stress in humans often leads to craving, and craving, in turn, frequently results in relapse. Thus, one can reasonably assume that opiate antagonists—which are considered anticraving medications—could reduce stress-induced craving and thereby decrease the risk of a stress-induced relapse. Although human studies have confirmed that naltrexone and another opiate antagonist, nalmefene, are both effective in preventing relapse in abstinent alcoholics (Schuckit 1996), the effects of opiate antagonists on stress-induced relapse have not yet been investigated specifically in humans.

### Effects of Alcohol Exposure on the Response to Stress

Not only can exposure to stress induce AOD self-administration in animals, but previous alcohol exposure influences an animal’s response to stress. In a study investigating the relationship between alcohol and stress, both alcohol-treated and control rats were repeatedly stressed by restraining their free movement for 2 hours daily for 5 days (Haleem 1996). The alcohol-treated rats in that study received alcohol in their drinking water for 2 weeks before being exposed to the restraint stress and continued to receive alcohol during the stress period. A single 2-hour restraining period on the first day of the experiment decreased food intake in both the alcohol-treated and the control rats. On the second and third day of the experiment, however, the control rats showed smaller decreases in food intake, and on the fifth day their food intake had returned to normal levels, suggesting that the animals had adapted to the stress. Among the alcohol-treated rats, however, the decrease in food intake was slightly attenuated after the second-day restraint but did not decrease further on the remaining days of the experiment. These findings suggest that alcohol exposure interfered with the rats’ ability to adapt to repeated stress.

In summary, researchers have extensively investigated the effects of stress on AOD self-administration in animals. These studies found that stressful experiences—whether acute or chronic and whether physical or psychological in nature—can contribute significantly to the animals’ AOD self-administration.

## The Relationship Between Stress and Alcohol Use in Humans

Consistent with the animal studies described in the previous section, clinical studies indicate that both acute and chronic stress may play a role in the development of AOD use disorders, the initiation of AOD abuse treatment, and the precipitation of relapse in recovering alcoholics. These three areas are discussed in the following sections.

### Stress and the Development of Alcoholism

Clinical and naturalistic studies have assessed the influence of both acute and chronic stress on drinking behavior and the development of alcoholism. Many of those investigations have focused on occupational stress as an example of chronic stress. For example, Seeman and Seeman (1992) found in a survey of more than 500 men that drinking problems were closely related to stressful experiences—whether they resulted from acute and severe stressors (e.g., illness or death of a loved one) or from chronic occupational stressors—that were combined with a strong sense of powerlessness. With respect to occupational stress, men in positions combining little freedom in choosing how to fulfill their job obligations (i.e., low job latitude) and high job demands reported the highest drinking levels and most alcohol-related problems.

The extent to which job stress influences drinking behavior also depends on the type of stress experienced. Thus, Crum and colleagues (1995) found that men employed in high-strain jobs (i.e., jobs with high demands and low control) generally had a higher risk of developing alcohol use disorders when compared with men in low-strain occupations (i.e., jobs with low demands and high control). However, this increase was greater for men in positions with high physical demands (three to four times higher risk) than for men in positions with high psychological demands (two to three times higher risk). Other studies noted that chronic, low-level, work-related stressors (e.g., uncooperative coworkers or daily parking problems) also were associated with higher drinking levels (Takeshita et al. 1998).

Several studies have focused specifically on the relationship between stress and alcohol consumption in women. Such analyses are of particular interest, because women may be more susceptible than men are to some of alcohol’s harmful health effects (Lex 1991). Furthermore, women have been reported to be more likely than men to consider stressful events as being associated with the initiation of problem drinking (Lex 1991). The latter association was not confirmed, however, in a critical review of stressful life events and drinking behavior in women. In that review, Allan and Cooke (1985) found no evidence of a gender-specific relationship between stress and alcohol abuse in women, although the researchers noted a high prevalence of stressful life events (e.g., divorce or death of a loved one), particularly among middle-aged women who developed alcohol dependence later in life. Most studies reviewed, however, failed to address the possibility that heavy drinking may be the cause rather than the consequence of life stressors.

Although the general association between stress and drinking behavior in women has remained controversial, some studies have found an important relationship between women’s coping styles and stress-related alcohol consumption. In those studies, women who used problem-focused coping strategies (i.e., who took specific measures to eliminate or address the source of the stress) consumed less alcohol during stressful periods in their lives than did women who used coping strategies that focused on emotions or which merely served to relieve the immediate negative emotions (i.e., were palliative) rather than address the problem (Breslin et al. 1995). Accordingly, treatment modules teaching problem-focused coping skills may be an important component of effective therapy for some AOD-abusing clients.

Another approach to investigating the role of stress in the development of alcoholism has been to analyze alcohol’s stress-response dampening (SRD) effects in different populations. SRD effects are those consequences of alcohol consumption that result in a reduction of both the body’s emotional responses (e.g., anxiety, tension, and nervousness) and physiological responses (e.g., changes in heart rate or sweating) to stress. (For more information on alcohol’s SRD effects, see the article in this issue by Sayette, pp. 250–255.) Sher and Levenson (1982) found that alcohol’s SRD effects were more pronounced in nonalcoholic people who demonstrated personality traits that have been associated with a risk for the development of alcoholism (e.g., aggressiveness, impulsivity, and outgoing than in people without those characteristics. The researchers suggested that because of their enhanced SRD experience, people with those personality traits were likely to find alcohol consumption particularly reinforcing, increasing their risk for alcoholism. More recently, Sinha and colleagues (1998) determined that women with a family history of alcoholism or anxiety disorders, who are at increased risk for alcoholism, exhibit a greater SRD effect of alcohol than do women without such a family history. Again, it is an intriguing notion that this population has an increased risk of alcoholism, because alcohol may be particularly reinforcing as the result of its potent SRD effect. Thus, these studies suggest that an enhanced sensitivity to alcohol’s SRD effect may contribute to an increased vulnerability of people with anxiety disorders for initiating and escalating alcohol use.

### Stress and Treatment Initiation

Discrete stressful events often provide impetus to an alcoholic person to seek treatment, especially when other resources and responses have failed to alleviate the stressful situation. This correlation between stress and treatment initiation was highlighted in several studies comparing alcoholics who had initiated treatment with alcoholics who received no treatment. In those comparisons, alcoholics entering treatment were more likely to perceive their drinking problems as severe, had more symptoms of alcohol dependence, and experienced more stressors and negative events in various life domains (Finney and Moos 1995). Of prime importance, these stressors included both chronic hardships (e.g., strains in employment or marriage) and acute stressful events (e.g., accidents, criminal charges, or divorce) that often are associated with drinking.

Alcoholics with greater resources in multiple domains (e.g., those who are employed and have an intact marriage) are likely to seek treatment for alcohol-related problems more quickly than are alcoholics with fewer resources. For example, social resources, such as an extended network of family members and friends, may increase the probability that a drinker’s alcohol-related problems are pointed out to him or her by other people, thereby leading to early treatment seeking. This hypothesis contradicts the notion that an alcoholic must lose all his or her resources (i.e., “ hit bottom” ) before seeking treatment; rather, it suggests that resources should be increased (“the bottom should be raised” ) so that the person seeks treatment before experiencing multiple devastating consequences of alcoholism.

In summary, stress in many cases may play a causal role in the initiation of treatment. This role, however, probably is moderated and mediated by numerous factors, including a drinker’s resources, social pressure, problem-solving skills, and coping strategies (Finney and Moos 1995).

### Stress and Relapse

Both discrete, stressful life events and chronic stressors may play a role not only in the development of alcoholism and AOD treatment initiation, but also in the relapse of people recovering from AOD abuse. To explain the association between stress and relapse, as well as the fact that not all AOD abusers relapse when encountering stress, Brown and colleagues (1990) have proposed the stress-vulnerability hypothesis. This hypothesis posits that AOD use in the face of severe stressors is mediated by the presence or absence of both protective factors (e.g., good social support) and risk factors (e.g., homelessness and unemployment). The hypothesis is supported by findings that severe stress (defined as life adversity posing either a high personal threat or chronic coping demands) which occurred prior to and independent of alcohol use was related to relapse after treatment (Brown et al. 1990). Thus, during a 3-month followup period after treatment, patients who relapsed had experienced twice as much severe stress before entering treatment compared with patients who remained abstinent. The study also calculated a composite “psychosocial vulnerability score” based on the patient’s coping skills, social resources, confidence that he or she would be able to resist an urge to drink, and level of depression. According to that analysis, people whose scores in these areas improved during treatment had better outcomes (i.e., a lower risk of relapse). These findings emphasize the connection between stress and relapse and suggest that resilience to stress-induced relapse can be improved during treatment.

Another study followed a large group of alcoholics, opiate users, and cigarette smokers in early abstinence to investigate the effects of acute stress and commitment to abstinence on relapse (Hall et al. 1990). The commitment to abstinence was measured using a scale that allowed the participants to choose between six different treatment goals, ranging from abstinence to no change in use. The researchers found that commitment to abstinence was the strongest predictor of abstinence during the followup period. Furthermore, an association between elevated stress levels and relapse existed only when the subjects were interviewed *after* their relapse (i.e., retrospectively) about the factors contributing to their relapse, but not when stress levels were assessed *before* a relapse occurred (i.e., prospectively). This observation suggests that stress may not actually lead to relapse; instead, the relapse may have resulted in increased stress and the subjects may have used the attribution of stress as causing the relapse as a way to make sense of the relapse. The actual relationship between stress and relapse in this study is difficult to assess, however, because the followup period was rather brief (i.e., 12 weeks) and the study did not assess the effects of chronic stress. Nevertheless, the study results emphasize the need for more careful, prospective studies of the relationship between stress and relapse.

## Stress Management in AOD Abuse Treatment

As the studies reviewed in the previous section indicate, stress may play a crucial role in the relapse to AOD abuse after treatment. Accordingly, the incorporation of treatment strategies to help patients cope with stressful events could reduce relapse risk. Such strategies may include pharmacotherapeutic as well as psychosocial approaches, as discussed in the following sections.

### Pharmacotherapy

Besides contributing to relapse to alcohol, stress also may play a role in relapse to other psychiatric disorders, such as depression and anxiety. As with the treatment of such disorders, it therefore makes sense that pharmacological management for recovering AOD users should be maximized during times of stress to help reduce risk of relapse. Accordingly, treatment of anxiety may be a useful component of alcoholism treatment.

As mentioned earlier in this article, at a neurochemical level the connection between stress (and/or anxiety) and resumption of alcohol use appears to involve several neurotransmitter systems in the brain, including serotonin pathways and reward pathways, which use dopamine and opioid peptides. Accordingly, medications affecting those systems (e.g., SSRIs and opioid antagonists) may play a significant role in minimizing the risk of relapse after stressful events. Other anxiety-reducing therapeutic agents that act on the same systems, such as benzodiazepines, have abuse potential themselves, making their use in people with AOD use disorders risky.

SSRIs may have particular appeal for use in AOD users, because these agents are easy to take (e.g., require only one daily dose), have no abuse potential, and are relatively safe when used in excessive doses or combined with AODs. Higley and colleagues (1998) examined the efficacy of the SSRI sertraline in mitigating the effects of stress on alcohol consumption in nonhuman primates. In that study, sertraline reduced alcohol consumption and aggressiveness in animals that had been exposed to stress before being returned to their home cages, presumably by increasing serotonin activity in the central nervous system. Under conditions of extreme stress, however, sertraline became ineffective and the animals resumed high drinking levels.

Based on those observations, the investigators suggested that relapse to drinking may be most likely to occur during periods of stress and that SSRIs may not be able to prevent such a relapse. Under nonstress conditions, including following a stressful situation, however, SSRIs, such as sertraline, may be effective in reducing alcohol consumption. Thus, pharmacological treatment with SSRIs may be most effective in conjunction with other nonpharmacological therapies, such as cognitive-behavioral therapy, for improving or preventing stress. Interestingly, in a study among patients with posttraumatic stress disorder (PTSD), patients treated with the SSRI fluoxetine (Prozac^®^ ) showed improvement on a scale designed to measure stress resilience (Connor et al. 1999).

The opioid antagonist naltrexone also has been shown to be effective in preventing relapse in detoxified alcoholics (O’Malley et al. 1992; Volpicelli et al. 1992). These effects result at least in part from the agent’s effect on reward pathways. In animal models opioid antagonists also have been shown to prevent increased drinking in animals that had experienced a stressful situation, suggesting that these medications also may influence the stress-drinking correlation (Volpicelli et al. 1986). However, researchers have not yet studied systematically this potential role of naltrexone.

### Psychosocial Therapy

If stress, the vulnerability to stress, and the presence or absence of protective factors are important mediators in the initiation of and relapse to AOD use, then specific treatment approaches targeting these areas might play a central role in the prevention and treatment of AOD use. Indeed, most AOD treatment approaches currently used contain social skills training and problem-solving components. This treatment philosophy is based on the classic relapse prevention model proposed by Marlatt and Gordon (1985), which features such important stress management components as cognitive restructuring, coping skills, and problem-solving skills. For example, cognitive restructuring teaches people to interpret events, attitudes, and feelings in a rational way and to respond constructively to a crisis or stressful situation. Similarly, problem-solving skills training teaches patients to analyze problem situations and act constructively rather than impulsively. Finally, most treatment programs recognize the importance of social support systems in managing stress and therefore encourage patients to attend such self-help groups as Alcoholics Anonymous and Narcotics Anonymous and/or to recruit support from friends and family.

Although such a stress management approach has intuitive appeal, researchers have not thoroughly explored and evaluated this approach experimentally. For example, whereas numerous studies have demonstrated the usefulness of relapse-prevention protocols using these and other strategies in the treatment of AOD abuse, weight loss, and smoking cessation, many of those studies could not assess the specific contributions of the stress-reduction techniques to recovery.

A few studies, however, have attempted to identify the effective components of therapy. For example, in one study of 130 AOD abusers, patients who received supplemental skills training and social network development after treatment showed greater improvement in several areas (e.g., avoidance of AOD use; problem-solving; and coping with stress, relapse, and social interaction) compared with a control group (Hawkins et al. 1986). In a study of coping responses and relapse in adolescents, lower stress management abilities (e.g., fewer problem-solving and/or coping strategies and less self-assurance) were associated with a higher risk of relapse (Myers and Brown 1990). In another study of relapse among adolescent AOD users, self-esteem, high quality of support, and social support satisfaction—all of which are likely to improve stress vulnerability—were crucial determinants of a subject’s outcome at 6 months (Richter et al. 1991).

In summary, stress management techniques are an integral part of most AOD abuse treatment programs, although it is difficult to specifically ascertain the value of these techniques. Studies that have attempted to examine this issue, however, have demonstrated that measures both to enhance healthy coping strategies and problem-solving techniques and to maximize social support systems are important components of successful treatment.

## Summary

The relationship between stress and AOD use is complex. Most likely, however, stress and the body’s response to it do play a role in the vulnerability to initial AOD use, initiation of AOD abuse treatment, and relapse in recovering AOD users. This relationship probably is mediated, at least in part, by common neurochemical systems, such as the serotonin, dopamine, and opiate peptide systems, as well as the HPA axis. Further exploration of these connections should lead to important pharmacological developments in the prevention and treatment of AOD abuse, including alcohol use disorders.

Effective psychosocial approaches to AOD abuse treatment all contain elements aimed at reducing and managing stress. It is difficult, however, to separate the effects of such specific stress-reduction techniques from the effects of other effective treatment components. Nevertheless, studies indicate that treatment techniques that foster coping skills, problem-solving skills, and social support play a pivotal role in successful treatment. In the future, individualized treatment approaches that emphasize stress management strategies in those patients in whom a clear connection between stress and relapse exists will become particularly important.

## Footnotes

1 Naturalistic studies are investigations that are not conducted in a clinical setting.

2 The motivational, reinforcing, or rewarding effects of a drug (e.g., euphoria) are those effects that motivate the drug user to seek the “reward” and therefore consume more of the drug.
